# Bilateral Pheochromocytoma and Paraganglioma Tumors Due to Von Hippel-Lindau Syndrome in a 15-Year-Old Boy: A Case Report

**DOI:** 10.7759/cureus.47787

**Published:** 2023-10-27

**Authors:** Saeed Khalaf, Hasan F Jamal, Zahra S Alawi, Mahmood Alsaeed

**Affiliations:** 1 Internal Medicine, Salmaniya Medical Complex, Manama, BHR; 2 Geriatrics, Salmaniya Medical Complex, Manama, BHR

**Keywords:** secondary hypertension, bilateral adrenal adenoma, von hippel-lindau, paraganglioma, pheochromocytoma

## Abstract

Pheochromocytomas and paragangliomas are catecholamine-secreting tumors that originate from chromaffin cells of the adrenal medulla and autonomic neural ganglia, respectively. Patients with pheochromocytomas and paragangliomas typically present with paroxysmal headache, sweating, tachycardia, and hypertension. Although most pheochromocytoma cases are sporadic, many of the cases occur as part of a genetic disorder. Here, we report the case of a 14-year-old male patient who presented with hypertension. Laboratory tests showed elevated levels of serum and urinary catecholamines, metanephrines, and chromogranin. Abdominal ultrasound and computed tomography studies revealed bilateral solid adrenal masses and an isolated splenic mass. Further assessment identified an underlying Von Hippel-Lindau syndrome. The patient was initially treated medically and later surgically. This case highlights the importance of always considering pheochromocytomas and paragangliomas as rare differentials of secondary hypertension, especially in the presence of episodic headaches, sweating, and tachycardia. Furthermore, screening for underlying genetic disorders, such as in our case, should be considered in cases of bilateral tumors, onset at a young age, and presence of extra-adrenal tumors.

## Introduction

Catecholamine-secreting tumors are uncommon causes of hypertension [1]. Generally, they are classified into two main types including pheochromocytomas which originate from chromaffin cells of the adrenal medulla and paragangliomas, which arise from the extra-adrenal autonomic paraganglia [1]. Although both types of tumors have similar clinical features and management approaches, the distinction between them is crucial to screening for the associated neoplasms and gene mutations [2]. Epidemiological studies revealed that the global incidence of pheochromocytoma is between two and eight cases per million and the prevalence ranges between 1:2500 and 1:6500 [3]. Among patients with hypertension, the prevalence of pheochromocytoma ranges between 0.1-0.6% and 2.0-4.5% in adults and children, respectively [3].

Patients with pheochromocytomas and paragangliomas can be asymptomatic or present with variable non-specific symptoms depending on the location and size of the tumor; hence, they are often referred to as the “great mimickers” [2]. If present, the clinical features of pheochromocytomas and paragangliomas are due to the hypersecretion of catecholamines such as norepinephrine, epinephrine, and dopamine. These features include the classic triad of paroxysmal headaches, sweating, and tachycardia. However, the classic triad presents in less than 50% of the cases [4]. Additional symptoms include palpitations, blurred vision, chest pain, tremors, nausea, and vomiting. Hypertension is the most common sign that might be either sustained or paroxysmal [4].

Compared to adults, pediatric patients with pheochromocytomas are more likely to have an underlying genetic or familial disease, bilateral adrenal tumors, multiple tumors, and extra-adrenal tumors [2]. For instance, children with pheochromocytomas are three to four times more likely to have extra-adrenal neoplasms than adults and three to seven times more likely to have multiple tumors than adults [5]. Although most pheochromocytoma cases are sporadic, up to 40% of the cases occur due to genetic disorders [2]. Several genetic disorders are associated with pheochromocytoma tumors including von Hippel-Lindau (VHL) syndrome, multiple endocrine neoplasia type 2 (MEN2), and less commonly, neurofibromatosis type 1 (NF1) [6]. All these disorders have autosomal dominant inheritance patterns. Approximately 15% and 50% of patients with VHL and MEN-2 syndromes are associated with pheochromocytomas or paragangliomas, respectively [6].

VHL syndrome is an autosomal dominant familial neoplastic syndrome caused by genetic aberrations of the tumor suppressor gene VHL. According to the genotype and phenotype, VHL is classified into two types: type 1 which occurs due to truncating or missense mutations and type 2 which occurs due to missense mutation [7]. Additionally, type 2 VHL is further subdivided into three subtypes: type 2A VHL (high risk of pheochromocytomas and low risk for renal cell carcinoma), type 2B VHL (high risk for renal cell carcinoma and pheochromocytomas) and type 2C (pheochromocytomas only) [7].

Here, we report the case of a 14-year-old male patient who presented with hypertension due to pheochromocytoma. Further assessment revealed an underlying VHL genetic disorder. This case reiterates the need for screening for pheochromocytomas in cases of hypertension in young patients with episodic symptoms of sweating and headaches. Moreover, this case emphasizes the need to perform genetic testing in cases of bilateral tumors and those occurring at a younger age.

## Case presentation

A 14-year-old boy with no underlying chronic illnesses presented to our tertiary care center with a six-month history of severe headaches and vomiting. The headaches were frontal, throbbing in nature, paroxysmal, progressive, and worsened over the last two weeks before the presentation. The patient had no other symptoms such as dizziness, loss of consciousness, neurological deficits, visual impairment, chest pain, dyspnea, or fever. His family history included a history of essential hypertension in both parents and otherwise healthy siblings.

The patient’s physical assessment revealed an elevated blood pressure (195/130 mmHg) consistent with bilateral readings. Cardiovascular, neurological, thyroid, and renal examinations were normal. Additionally, electrocardiography was normal. Initial laboratory investigations including complete blood count, renal function, troponin, electrolytes, and urine analysis were normal (Table 1). Given the severe headache and vomiting, a brain computed tomography (CT) scan was done to assess for intracranial space occupying lesions which showed no specific findings.

**Table 1:** Laboratory serum investigations on presentation

Test	Result (Reference range)
White blood cell count	9.5x10^9^/L (3.6 - 9.6)
Hemoglobin level	12.8 g/dL (12.0-14.5)
Red blood cell count	5.6 x10^12^/L (3.9-5.2)
Platelet count	768 x10^9^/L (150-400)
Urea	4.2 mmol/L (3.2-8.2)
Creatinine	43 μmol/L (44-88)
Estimated glomerular filtration rate	220 mL/min/1,73m^2^ (>90)
Troponin	0.01 ng/mL (<1.5)
Sodium	142 mmol/L (132-146)
Potassium	5.0 mmol/L (3.5-5.5)
Phosphorous	1.60 mmol/L (0.95-1.65)
Albumin	45 g/dL (32-45)

Based on the patient’s clinical picture of periodic severe headaches and hypertension, secondary hypertension was suspected. He was, therefore, admitted for further assessment and management. The patient was then started on a calcium channel blocker (nifedipine extended release 30 mg daily) with subsequent blood pressure readings ranging between 140/90 mmHg and 170/100 mmHg.

Further investigations to assess secondary causes of hypertension were conducted. These investigations included thyroid stimulating hormone, aldosterone, renin, random cortisol, aldosterone renin ratio, serum and urinary catecholamines, and metanephrine levels. Hormonal assays revealed elevated levels of serum and urinary catecholamines, metanephrines, and chromogranin (Table 2).

**Table 2:** Further investigations to identify secondary causes of hypertension

Test	Result (Reference range)
Serum hormones	
Thyroid stimulating hormone	3.11 mIU/L (0.25 -5.0)
Aldosterone (supine)	19.60 ng/dl (0.07-1.08)
Renin (supine)	49.9 μIU/ml (2.8-39.9)
Aldosterone renin ratio	0.39 ng/dl/ per μIU/ml (<3.7)
Morning cortisol (8 am)	630 nmol/L (193-690)
Urinary catecholamines	
Chromogranin	432 μg/l (<102)
Noradrenaline /creatinine	7970 μg/g crea (5.0-53.0)
Dopamine /creatinine	772 μg/g crea (59.0-552)
Metanephrine /creatinine	85 μg/g crea (≤228)
Nomtanephrine /creatinine	16791 μg/g crea (25-339)
Vanillylmandelic acid /creatinine	46.7 mg/g crea (1.0-5.6)
Serum catecholamines	
Noradrenaline	6120 ng/l (≤499)
Dopamine	316 ng/l (≤100)
Metanephrines	<50 ng/dl (<95)
Normetanephrine	>5000 ng/dl (<129)

**Figure 1:**
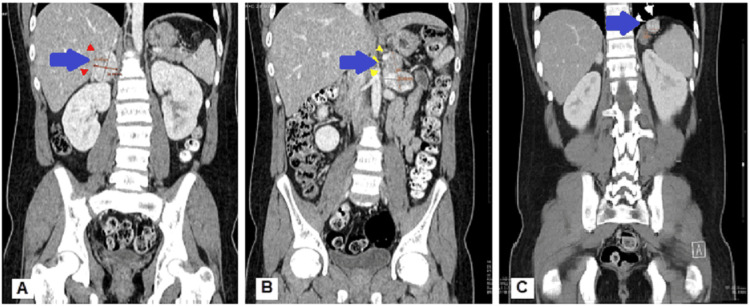
Abdominal CT images (coronal section) The coronal section of abdominal computed tomography shows a 42 x 34 mm right adrenal mass that is lobulated with exophytic enhancement, central hypodensities, and suggestive necrosis (A), a 43 x 27 mm left adrenal mass that is lobulated with exophytic enhancement and central hypodensities, suggesting necrosis (B), and a left-sided extra-adrenal lesion (C).

**Figure 2:**
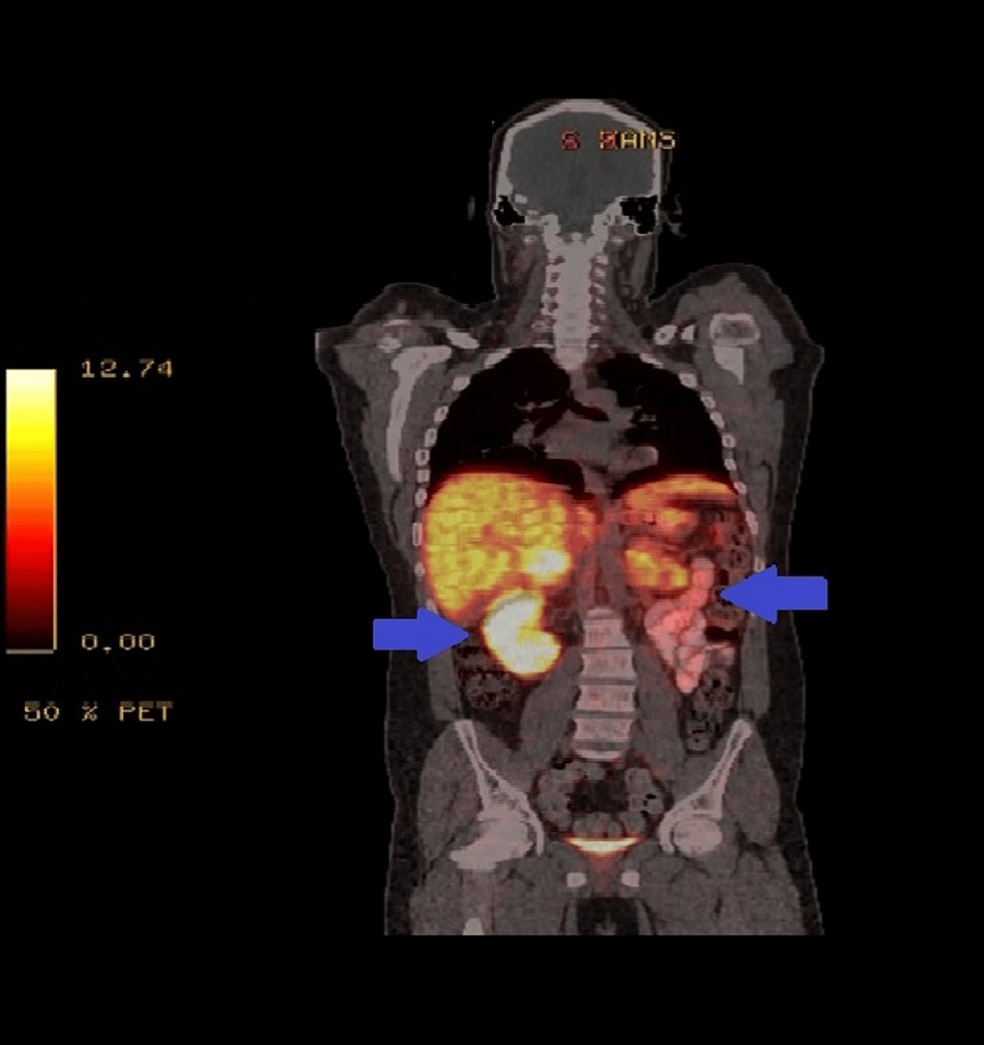
PET scan image of the abdomen Positron emission tomography scan showing bilateral abnormal increased uptake at both right and left adrenal glands (blue arrows).

In view of the elevated renin and catecholamines, an abdominal and renal ultrasound study was conducted to assess the adrenal glands, renal arteries, and kidneys. Ultrasonography showed bilateral solid adrenal masses and the abdominal CT revealed bilateral 42 x 34 mm and 43 x 27 mm adrenal masses that were lobulated with central hypodensities and exophytic enhancement. In addition to that, another 22 x 21 mm mass was found in the left subphrenic space (Figure 1). A positron emission tomography (PET) scan was also conducted which showed no distant metastasis (Figure 2).

**Figure 3:**
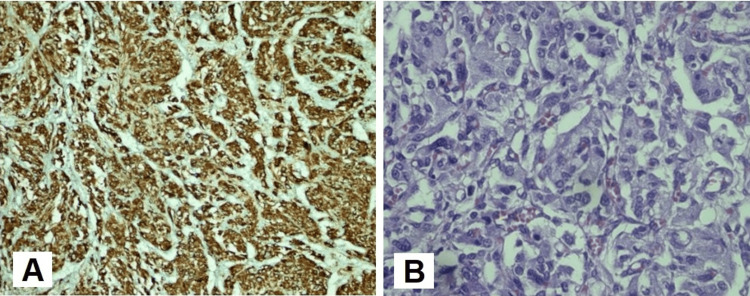
Histopathological assessment of the excised specimen Histopathological assessment of the excised specimen shows diffuse chromogranin positive cells (A) and cells with abundant fine, granular amphiphilic cytoplasm with mostly uniform round to oval nuclei and prominent nucleoli (B).

Based on the clinical picture, laboratory tests, and radiological investigations, a diagnosis of bilateral pheochromocytoma with a splenic lesion was made. Thus, α and β-adrenergic blockers were started including once daily metoprolol 25 mg and prazosin 1 mg tablets. Clinically, the patient’s symptoms improved dramatically with medical treatment. Subsequently, he underwent laparoscopic bilateral sub-total adrenalectomy and left-sided subdiaphragmatic mass excision. The pathological examination of the masses confirmed the diagnosis of pheochromocytoma and paraganglioma (Figure 3).

The patient’s postoperative course was uneventful and blood pressure readings returned to normal levels even after discontinuation of all antihypertensives. Maintenance oral hydrocortisone was also started (20 mg in the morning and 10 mg in the evening). Genetic testing was positive for VHL syndrome. Thus, screening for the patient’s family members was done.

## Discussion

Here, we presented a rare case of pheochromocytoma and paraganglioma due to underlying VHL syndrome. Our patient presented with typical symptoms of paroxysmal hypertension and headaches. The differential diagnoses of recurrent spells of palpitations, headaches, hypertension, and sweating include pheochromocytoma, hyperthyroidism, hyperadrenergic spells, hypoglycemia, postural orthostatic tachycardia syndrome, mast cell disease, and carcinoid syndrome [8]. Our case highlights the need to consider such diagnoses in patients with a similar presentation especially given the patient’s age.

The diagnostic work-up of pheochromocytomas and paragangliomas includes biochemical hormonal detection of excess catecholamine secretion, imaging studies to locate and stage the tumor, and genetic testing. Additional tests might be needed to detect syndromic features if the diagnosis of a genetic syndrome is confirmed. The initial biochemical testing should include plasma-free metanephrines or urinary fractionated metanephrine levels (sensitivity of 99% and 80%, respectively) [9]. Patient’s position, caffeine consumption, nicotine, alcohol, chocolate, and medications like monoamine oxidase inhibitors, ephedrine, tricyclic antidepressants, serotonin reuptake inhibitors, morphine, levodopa, methyldopa, and buspirone might interfere with the results and cause false positive results [9]. Thus, it is crucial to consider these specific measures during the assessment.

The biochemical assessment should be followed by imaging studies to localize and stage the tumors. CT scan is the recommended imaging modality as it provides better spatial resolution than magnetic resonance imaging (MRI) [9]. In addition to that, CT is recommended for patients with positive biochemical tests. In our case, a CT was performed after biochemical tests indicated a possibility of a pheochromocytoma. In CT scans, these tumors appear as well-circumscribed solid hypervascular masses that range between 1 and 15 cm. MRI is the preferred modality for pediatric patients, pregnant patients, and patients with allergic reactions to iodinated contrast medium [10]. Additionally, several functional imaging tests are highly sensitive and specific and can be used to diagnose, stage, and follow up patients with pheochromocytomas or paragangliomas including scintigraphy and PET [9]. Nuclear imaging should be reserved for large tumors (>8 cm), paragangliomas, adrenal pheochromocytomas, and if CT or MRI studies are negative despite the high suspicion of pheochromocytoma [10].

Genetic testing should also be considered in all cases of pheochromocytomas and paragangliomas; particularly, in pediatric patients, patients with bilateral or extra-adrenal tumors, and those with known genetic disorders [11]. Around 5% of adrenal incidentalomas are due to pheochromocytomas and testing for pheochromocytoma should be considered in masses that are cystic, vascular, and have equal or more than 10 Hounsfield unit attenuation in non-contrast CT scans [12,13].

A multidisciplinary approach that considers patient characteristics like age, physical status, underlying comorbidities, tumor features such as primary tumor site, presence of metastasis or tumor growth rate, and genetic profile is needed to manage pheochromocytomas and paragangliomas [14]. Surgical resection remains the mainstay therapy for majority of the tumors [14]. In our case, symptoms resolved after surgical resection. Catecholamine blockade through α-adrenergic receptor blockers is recommended to manage hypertension and to prevent perioperative cardiovascular complications that might result from a massive surge release of catecholamines [15]. Additionally, beta receptor antagonists may be used to minimize secondary tachycardia associated with the use of nonselective alpha-blockers, but only after administration of α- adrenergic receptor blockers due to the risk of hypertensive crisis and pulmonary edema [15].

Follow-up visits to assess the clinical and biochemical response to treatment are recommended. The follow-up visit should include an assessment of blood pressure, catecholamine biomarkers, imaging studies, and screening for associated cancers such as renal and brain tumors and retina screening if a genetic disorder is confirmed [16]. Our patient had complete resolution of symptoms with normal blood pressure readings at follow-up. The overall prognosis for pheochromocytomas and paragangliomas depends on the stage of the tumor, the presence of metastasis, and the associated genetic disorders. Young age, local tumor, female sex, underlying genetic disorders, and asymptomatic tumors are associated with better prognosis [17].

## Conclusions

Pheochromocytomas and paragangliomas are rare causes of secondary hypertension but should be suspected in certain circumstances, such as in our case. These include the presence of episodic headaches, sweating, tachycardia, and hypertension. Screening for underlying genetic disorders and genetic counselling should be considered in cases of bilateral tumors, pediatric patients, and extra-adrenal tumors. Surgical resection is the mainstay treatment, and regular follow-up is crucial to identify recurrence or missed lesions.
